# *Drosophila* Motor Neuron Retraction during Metamorphosis Is Mediated by Inputs from TGF-β/BMP Signaling and Orphan Nuclear Receptors

**DOI:** 10.1371/journal.pone.0040255

**Published:** 2012-07-05

**Authors:** Ana Boulanger, Morgane Farge, Christophe Ramanoudjame, Kristi Wharton, Jean-Maurice Dura

**Affiliations:** 1 Neurogenetics and Memory, Department of Genetics and Development, Institute of Human Genetics, CNRS UPR1142, Montpellier, France; 2 Division of Biology and Medicine, Department of Molecular Biology, Cell Biology and Biochemistry, Brown University, Providence, Rhode Island, United States of America; Columbia University, United States of America

## Abstract

Larval motor neurons remodel during *Drosophila* neuro-muscular junction dismantling at metamorphosis. In this study, we describe the motor neuron retraction as opposed to degeneration based on the early disappearance of β-Spectrin and the continuing presence of Tubulin. By blocking cell dynamics with a dominant-negative form of Dynamin, we show that phagocytes have a key role in this process. Importantly, we show the presence of peripheral glial cells close to the neuro-muscular junction that retracts before the motor neuron. We show also that in muscle, expression of *EcR-B1* encoding the steroid hormone receptor required for postsynaptic dismantling, is under the control of the *ftz-f1/Hr39* orphan nuclear receptor pathway but not the TGF-β signaling pathway. In the motor neuron, activation of *EcR-B1* expression by the two parallel pathways (TGF-β signaling and nuclear receptor) triggers axon retraction. We propose that a signal from a TGF-β family ligand is produced by the dismantling muscle (postsynapse compartment) and received by the motor neuron (presynaptic compartment) resulting in motor neuron retraction. The requirement of the two pathways in the motor neuron provides a molecular explanation for the instructive role of the postsynapse degradation on motor neuron retraction. This mechanism insures the temporality of the two processes and prevents motor neuron pruning before postsynaptic degradation.

## Introduction

During development, the dendrites and axons of specific neurons are eliminated without cell death. This phenomenon of neuronal remodeling (pruning) is conserved throughout evolution and is present in both vertebrates and invertebrates. Importantly, the pruning of dendrites and axons also occurs in the adult in response to injury or disease [1]. Neuronal remodeling provides a mechanism for developmental neural plasticity in a maturing nervous system. This developmental plasticity is considered necessary for the normal function of the mature nervous system although direct proof for such a requirement is sparse. Axon pruning occurs through two different mechanisms: either by retraction or degeneration. *Drosophila* mushroom body (MB) γ neuron remodeling is a well-documented case of axon degeneration [2]. In insects, maturation at metamorphosis is regulated by the steroid hormone 20-hydroxyecdysone (ecdysone). Ecdysone binds ecdysone receptor (*EcR*), whose expression is essential for neuronal remodeling. Thus, *EcR-B1* expression is specifically up-regulated in MB γ neurons before a crucial pre-pupal ecdysone pulse, but not in MB neurons that do not remodel [3]. Expression of *EcR-B1* is also critical for the dendritic destruction of dendritic arborizing (da) sensory neurons that involves both local degeneration and branch retraction [4].

Two pathways appear to act independently to activate the transcription of *EcR-B1* in a cell-autonomous fashion. First, the TGF-β signaling pathway has been shown to act through type I and II receptors (*babo* and *punt/wit*) to phosphorylate the *dSmad2* protein which then shuttles to the nucleus, presumably to influence gene expression [5]. Recently, a second independent pathway was described where the orphan nuclear receptor FTZ-F1 activates *EcR-B1* expression and at the same time represses *Hr39* its homologous gene. If inappropriately expressed in the γ neurons, HR39 inhibits normal pruning, probably by competing with endogenous FTZ-F1, which results in decreased *EcR-B1* expression. Thus, several parallel pathways converge on the *EcR-B1* promoter and allow axon degeneration in selected neurons of the MBs [6].

During *Drosophila* metamorphosis, the motor neurons innervating the larval muscles at the neuromuscular junctions (NMJs) are also remodeled. Motor neuron remodeling parallels muscle dismantling. Indeed, the majority of larval musculature is histolyzed and replaced by the proliferation of adult muscle progenitors [7]. On the other hand, it is probable that most motor neurons survive metamorphosis and are structurally respecified to innervate adult muscle targets [8]. Little is known about the genetic and cellular control of the dismantling processes of *Drosophila* NMJ during metamorphosis [9]. Here we show that in muscle, *EcR-B1* expression required for postsynaptic dismantling, is under the control of the *ftz-f1/Hr39* nuclear receptor pathway. In the motor neuron, activation of *EcR-B1* expression, which triggers axon pruning, is mediated by TGF-β signaling and nuclear receptors. Our results clearly indicate that in this case it is axonal retraction that occurs and not degeneration. Importantly, glial cell and phagocytes seem also to have a key role in this process.

## Materials and Methods

### Fly Stocks and Genetics

*Drosophila melanogaster* lines were maintained at 25°C on standard sugar, cornmeal, yeast extract food except for crosses involving the *UAS-shi^ts1^* allele that were maintained at permissive temperature (18°C). The following strains were used in this study: *MHC-Shaker-mGFP* was a courtesy of A. DiAntonio (Washington University Medical School, St. Louis, MO) [10]. *MHC-GAL4* driver [11], *OK6-GAL4* (from C. O’Kane, University of Cambridge, Cambridge, UK) [12] and *BG380-GAL4 (*from V. Budnik, University of Massachusetts Medical School, Worcester, MA) [13] were also used. *y w^67c23^, UAS-mCD8-GFP* (2 transgenes*), UAS-lacZ, repo-GAL4*, *Coll-GAL4*, *Hml-GAL4* [14], alleles *wit^A12^* and *wit^B11^* [15], *UAS-EcRB1* and *UAS-EcR-B1^DN^ ΔC655-F645A* were from the Bloomington stock center (Bloomington, IN). *Pxn-GAL4, UAS-GFP* was from Paul Martin [16]. Recombinant *repo-GAL4, UAS-GFP* was provided by A. Garcés (INM, Montpellier, France). *UAS-baboRNAi* (on X), *UAS-MadRNAi* (on III) and *UAS-puntRNAi* (on III) were from NIG-Fly, Japan. Alleles *ftz-f1^17^* and *ftz-f1^19^* were a courtesy of C. T. Woodard (Mount Holyoke College, South Hadley, MA 01075) [17]. *UAS-wit^DN^ (*allele *wit^941^)* was provided by G. Marques (University of Alabama, Birmigham, AL). *UAS-wit^DN^* was recombined with *OK6-GAL4* to create the *UAS-wit^DN^, OK6-GAL4* strain. *UAS-baboΔI* (isoform a, 4 transgenes) [18] and the two different *puntΔI* named here 553 (2 transgenes on III) and 1-4 (2 transgenes on II), were from Michael O’Connor (University of Minesota, Minneapolis, MN) [19], *UAS-dad* was a courtesy of T. Tabata (Tokio University, Japon) [20]. We also used *UAS-Hr39* (on II), a double *UAS-Hr39; UAS-EcR-B1* strain, the recombinant *UAS-HR39, UAS-lacZ* [6] and the recombinant *OK6-GAL4, UAS-GFP.* In addition we used *UAS-shi^ts1^* [21], *UAS-Dicer2*; *MHC-GAL4* and *UAS-spectrin-Myc* (on III) from Greg Bashaw [22].

### Larval and Pupal Dissection

Third instar larval filets were performed in 1× PBS and fixed for 15 minutes in 4% formaldehyde in PBS. White pupae (0 h APF) were collected and placed at 25°C during the staging period except for white pupae containing the *UAS-shi^ts1^* allele and the corresponding controls which were placed at 29°C. Pupae were fixed for 1 hour in 4% formaldehyde or 15 minutes in Bouin’s solution when the anti-D-GluRIIC antibody was used. Larval ventral cords were fixed for 1 h in 2% paraformaldehydehyde when the EVE antibody was used.

### Immunohistochemistry

Samples were labeled as previously described [6]. The following antibodies were used: anti-DLG, (4F3), 1∶100; anti-Fasciclin II, 1D4, (1∶10); anti-cis string protein, (CSP), 1∶50; anti-Futsch, (22C10), 1∶50 (Developmental Studies Hybridoma Bank), Iowa (DHSB). Cy3 or Cy5-conjugated goat anti-HRP, 1∶100, (Jackson Immunoresearch laboratory); rabbit anti-D-GluRIIC, 1∶5000 (from A. diAntonio); rabbit β-Spectrin, 1∶500, (from R. Dubreuil); mouse anti-Acetylated Tubulin, 1∶600 (Sigma-Aldrich); mouse ECR-B1 (AD4.4 1:5,000) (from C. Thummel), rabbit Eve, 1∶3,000 (M. Frasch). Cy3 conjugated antibodies against rabbit or mouse IgG (1∶300, Invitrogen); Alexa 488 conjugated antibodies against mouse or rabbit IgG (1∶1000, Jackson Immunoresearch laboratory).

### Microscopy and Image Processing

Images were acquired using a Zeiss LSM 780 laser scanning Confocal microscope (MRI Platform, Institut of Human Genetics, Montpellier, France) equipped with a 40× PLAN APO 1,3 oil DIC objective lenses. The acquisition software was Zen 2010. Settings were optimized for detection without saturating the signal. For each set of figures settings were constants.

### NMJ Quantifications

For NMJ quantifications we used the ImageJ program. Images were z-stacked before processing. To calculate the motor neuron perimeter we outlined the motor neuron contour following the limits of the anti-HRP staining and then selected the ImageJ perimeter function. In order to evaluate the putative effect of total muscle size variations on these NMJ quantifications, we have determined the perimeter of muscle 4 at 2 h (889±12 µm and n = 9), 6 h (853±17 µm and n = 10) and 8 h (837±34 µm and n = 10) with a significance of P>0.1 in all the different combinations (between 2 h and 6 h, 2 h and 8 h and 6 h and 8 h). Since there are no statistically significant muscle perimeter differences, neither the motor neuron perimeter values nor the other NMJ quantifications (see below) were normalized with the muscle perimeter values.

To calculate the postsynaptic width we first placed a 35 µm line perpendicular to the second synaptic bouton (for 0 h, 2 h and 6 h when boutons are visible) or perpendicular to the motor neurons (for some 6 h and 8 h) in a merged anti-HRP and anti-DLG-stained image. Second we placed this line at the same level in the same anti-DLG-stained image and selected the ImageJ plot profile function. Next, in the resulting profile we measured the distance between peaks giving fluorescence values higher than 500. The bouton size was measured in a similar way using an anti-HRP stained image. In this case, we measured the distance between peaks giving fluorescence values higher than 1000. To determine the amount of debris we outlined the debris surrounding the NMJ and selected the ImageJ analyze particles function. We have considered as “arrested postsynaptic dismantling” a postsynapse in which the anti-DLG staining appears as a continuous structure surrounding the synaptic boutons and in which there are no visible filopodia. We have considered as “arrested presynaptic dismantling” a motor neuron (stained with the anti-HRP antibody) in which dendritic extensions contained at least two individual synaptic boutons connected by a link. For bouton number quantifications, type I_b_ boutons of muscle 4 on segment A3 were counted in *+/wit^DN^* and *wit^DN^*, *OK6/lacZ* L3 individuals. In addition, muscles in these individuals were photographed at 20× magnification and then traced and measured using ImageJ. The area of muscle 4 was similar (P = 0.695) in *+/wit^DN^* (42343±2473 µm^2^ and n = 10) and *wit^DN^*, *OK6/lacZ* individuals (40669±3397 µm^2^ and n = 10) at L3. Since no statistically significant muscle area differences were found, the number of boutons in each NMJ was not normalized with the muscle area values. To calculate the microtubule diameter we have traced a line 3 µm before the end of the anti-HRP staining at the distal tip of the motor neuron and perpendicular to the NMJ and measured the diameter of the microtubules based on the anti-Futsch staining.

### Quantification of ECR-B1 Immunolabelling

For ECR-B1 expression quantification we used composite of ten confocal images. Cell contours were drawn using ImageJ software. We performed measurements from the Cy3 channel in EVE-positive (or GFP-positive) motor neurons (Intensity 1, …25) and the same number of measurements in background. The mean of these background measurements is called the mean background for each stack. We then subtracted intensities of mean background for each intensity value (Intensity 1, …25– mean background) to obtain real intensity values of the two genetic conditions and compared them using statistical t-test. We assayed 5 ventral cords from controls and 5 ventral cords for mutants in total for experiments. We quantified five cells per stack. Experiments were scored blindly and this was the result of three independent stains.

### Statistics

Comparison of two groups expressing a quantitative variable were analyzed for statistical significance using two–tailed Student’s *t*–test and all error bars are expressed as±standard error of the mean (s.e.m.). Comparison between groups expressing a qualitative variable was analyzed using the χ^2^ test. Values of P<0.05 were considered significant. n represents hemisegments in all our quantifications.

## Results

### Motor Neuron Membrane Continuity May Reflect Retraction

**Figure 1:**
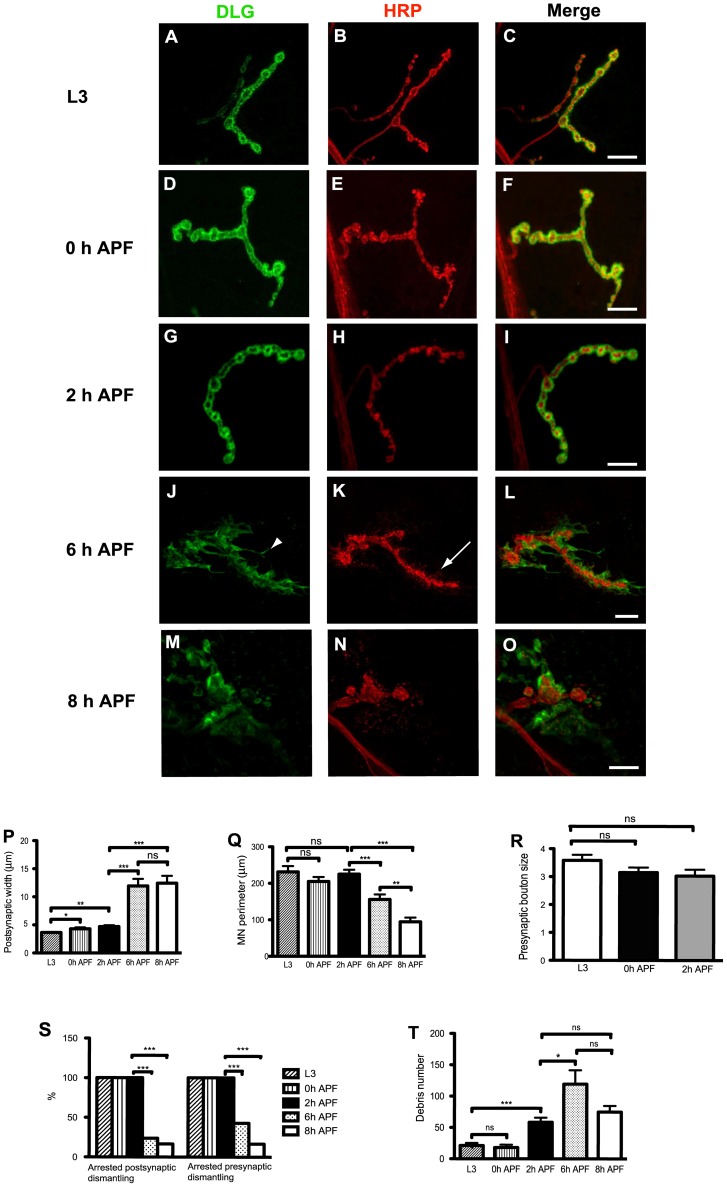
NMJ dismantling during *Drosophila* metamorphosis. **A-O**, Composite confocal images of muscle 4 NMJ in wild type larvae (L3) and pupae, labeled with anti-DLG antibody (green) and anti-HRP antibody (red). (A-C). At L3, the anti-DLG staining surrounds the bouton membranes as a thin layer. (D-I) Between 0 and 2 h APF the overall NMJ morphology was quite similar to L3. (J-L) 6 h APF the NMJ is disorganized, a high number of filopodia (arrowhead in J) and HRP-labelled debris were observed (arrow in K). (M-O) 8 h APF the anti-DLG staining became discontinuous and fragmented. Individual synaptic boutons are no longer observed with the HRP-staining. The motor neuron became shorter and swollen at the synapse. Bars, 20 µm. **P**, This histogram shows the width of the postsynapse. Results are means and s.e.m. n represents hemisegments in all our quantifications. L3 (n = 18), 0 h (n = 20), 2 h (n = 20), 6 h (n = 21) and 8 h (n = 23) APF. Note the statistically significant increase of the postsynaptic width at 0 h APF. **Q**, Histogram representing the perimeter size of the presynapse at different developmental stages. Results are means and s.e.m. The number of NMJ quantified was the following: L3 (n = 20), 0 h (n = 20), 2 h (n = 21), 6 h (n = 21) and 8 h (n = 24) APF. Note the statistically significant decrease in the motoneuron perimeter between 2 h and 6 h APF and between 6 h and 8 h. **R**, The histogram shows the diameter of the synaptic boutons (µm) at L3 (n = 19), 0 h (n = 18) and 2 h APF (n = 18). The synaptic boutons diameter was similar at the three stages analyzed. **S**, Percentage of post and presynapses displaying arrested dismantling at L3 (n = 20), 0 h APF (n = 21), 2 h APF (n = 13), 6 h APF (n = 21) and 8 h APF (n = 24). Both, the arrested post and presynaptic dismantling phenotypes decreased at 6 h APF. **T**, The number of debris particles was scored. L3 (n = 19), 0 h (n = 20), 2 h (n = 17), 6 h (n = 18) and 8 h (n = 19) APF. The amount of debris significantly increases at 2 h APF and then at 6 h APF. Results are means and s.e.m. The differences are significant in a t-test (*:P<0.05, **:P<0.01, ***:P<0.001) or not (ns: no statistical difference) in P-R and T. ***: P value <0.001 (χ^2^ test) in S. Genotype: *y w^67c23^.*

**Figure 2:**
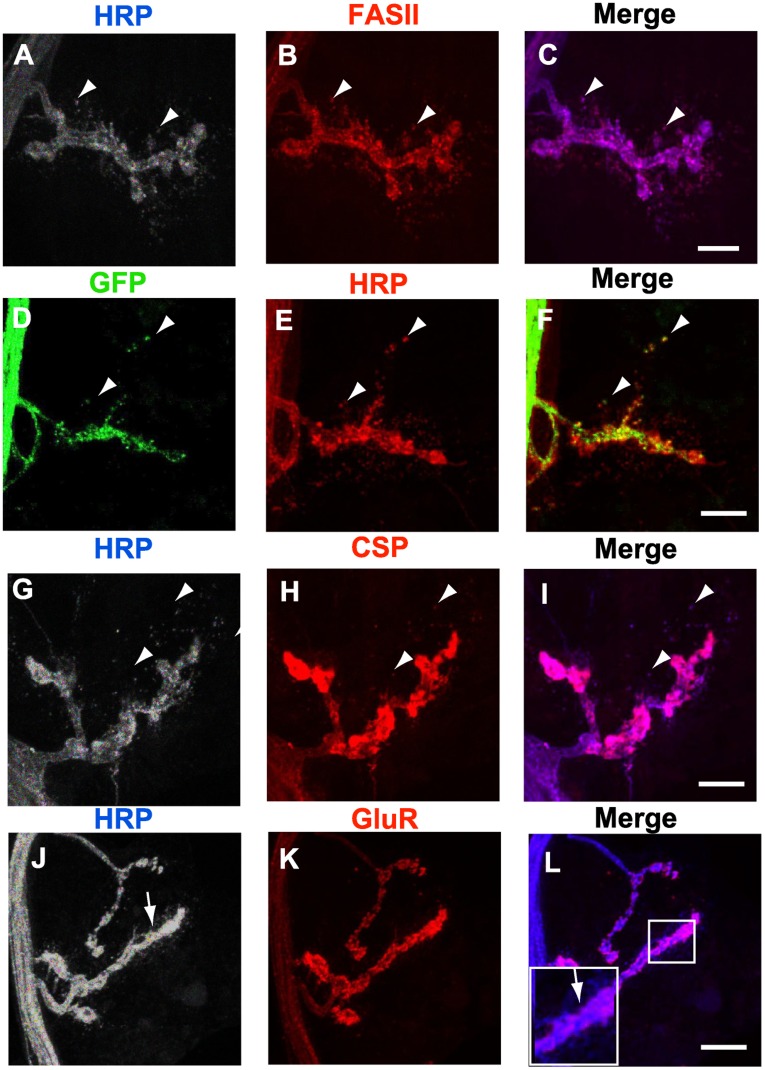
HRP-labeled debris are presynaptically derived. Composite confocal images correspond to 6 h APF pupae. **A-C**, The anti-HRP labeled puncta (grey, blue in the merge) colocalized with anti-FASII (red) (arrowheads). **D-F**, *UAS-mCD8-GFP* (green) was driven in motor neurons with *OK6-GAL4*. Most of the anti-HRP labeled debris (red) were GFP-positive (arrowheads). **G-I**, The anti-HRP labeled puncta (grey, blue in the merge) colocalized with CSP (red) (arrowheads). Note the pink color in the merge marked by arrowheads in C and I. **J-L**, The HRP-positive puncta (grey, blue in the merge) did not co-localize with D-GluRIIC (red), (arrow in J and L). The inset in L shows a high magnification (2×). Bars, 20 µm. Genotypes: (A-C, G-L) *MHC-mGFP-Shaker*, (D-F) *OK6-GAL4/2×UAS-mGFP.*

We examined the different steps of NMJ dismantling to determine whether motor neuron pruning was a mechanism of degeneration or retraction. Neuron membrane continuity would reflect retraction and neuron membrane disruption would reflect degeneration. We, thus, used HRP labeling of the motor neuron membranes to discriminate between degeneration or retraction. We analyzed specifically NMJ contacting muscle 4 at abdominal segment 3. Anti-HRP and anti-DLG staining of larval and pupal body walls showed well defined and organized synaptic boutons at this NMJ from L3 (Fig. 1 A-C), 0 hours after pupa formation (0 h APF) (Fig. 1D-F) and 2 h APF (Fig. 1 G-I). Conversely, by 6 h APF the NMJ appeared already completely disorganized (Fig. 1 J-L) thus preventing the distinction of individual synaptic boutons. Interestingly, we never observed disruptions of the parent motor neuron membranes with the HRP-staining, pointing to a retraction mechanism (Fig. 1). In addition, the postsynaptic components labeled with the anti-DLG antibody became fuzzy and the width of the DLG staining increased. By 8 h APF (Fig. 1 M-O) the DLG staining appeared completely fragmented and often absent and the presynapse as a shorter and swollen structure.

At 4 h, 5 h and 7 h APF intermediary NMJ dismantling was observed (Fig. S1 A-I). At 9 h and 10 h APF (Fig. S1 J-O) quite similar NMJ were observed. In this case, only small fragments labeled with DLG were visualized. HRP-labeled motor neurons contained often spherical and swollen structures or big debris detached from the parent axon. By 12 h APF (Fig. S1 P-R), NMJ became difficult to identify. We were able to determine the location of a particular muscle 4 NMJ only if DLG-labeled fragments were present or if an HRP-labeled presynaptic protrusion surrounded by debris appeared. Indeed, at this stage, the presynapse, when present, appeared as a short protuberance coming out of the nerve. HRP-labeled presynapse was never disrupted at these advanced pupal stages. Furthermore, we expressed a membrane tethered GFP *mCD8-GFP* in motor neurons using the *OK6-GAL4* driver, as a second motor neuron membrane marker, and analyzed presynaptic membrane morphology. As observed with the HRP-labeling, presynaptic membranes appeared as a continuous structure at 7 h, 10 h and 12 h APF (Fig. S2 A-C).

We have measured the variation of the postsynaptic width at different developmental stages **(** Fig. 1 P). A subtle but significant increase of the postsynaptic width was observed from L3 (3.65±0.19 µm) to 0 h (4.31±0.24 µm) and 2 h (4.71±0.23 µm) APF. A major increase took place between 2 h APF and 6 h APF (11.95±1.25 mm) as well as 8 h APF (12.43±1.31 µm). In order to describe precisely the observed decrease of motor neuron size, we have measured the perimeter of the HRP-labeled motor axons at the same developmental stages (Fig. 1 Q). No major changes on the perimeter length were observed from L3 (231.4±15.8 µm), 0 h (205.2±12.2 µm) and 2 h APF (225.2±12.5 µm). In contrast, at 6 h APF a statistically significant decrease of the perimeter length (155.8±13.9 µm) was observed. Furthermore, the motor neuron perimeter size decreased further by 8 h APF (94.6±11.6 mm). The moderate but statistically significant postsynaptic width increase from L3 to 0 h APF suggested that dismantling of the postsynaptic components was the first event of NMJ dismantling. In order to test further the hypothesis that postsynaptic events came before presynaptic bouton changes, we measured the diameter size of the synaptic boutons at these stages (Fig. 1 R). We found no significant variation of the synaptic boutons size from L3 (3.58±0.2 µm) to 0 h (3.15±0.18 µm), or 2 h (3±0.23 µm) APF. We have quantified the percentage of post and presynapses showing arrested dismantling (see materials and methods) at the same developmental stages (Fig. 1 S). The diminution of this percentage between 2 h APF and 6 h APF is clearly correlated to the increase of the postsynaptic width and decrease in motor neuron perimeter. Consequently, we have decided to restrict our quantification to the percentage of post and presynapses showing arrested dismantling for further experiments.

Interestingly, we have noticed the presence of HRP-immunoreactive debris invading the postsynapse and disconnected from the parent motor axon (arrow in Figure 1 K). HRP-immunoreactive debris have already been described at the NMJ in larval stages [23], as a result of NMJ growing arbors but never in pupa in association with neuronal retraction. We examined the amount of HRP-labeled debris (Fig. 1 T). Our analysis revealed that, even though some debris were seen at L3 (21±3.9), no significant change was observed by 0 h APF (18±4.5) but a significant increase was apparent by 2 h APF (58±7.3). A further increase in the amount of debris was observed at 6 h APF (119±22.2). Based on this data, it appears that the first sign of motor neuron pruning is debris shedding. By 6 h APF the HRP-labelled puncta perfectly co-localized with the adhesion molecule Fasciclin II (FASII) (Fig. 2 A-C) that has been described [11] to localize with both the pre and the postsynaptic components. Consequently, in order to determine the synaptic nature of the debris we first expressed a membrane targeted green fluorescent protein (*UAS-mCD8-GFP*) in motor neurons using the specific GAL4 driver *OK6-GAL4*. We observed that the HRP-labeled debris co-localized with the presynaptic GFP signal (Fig. 2 D-F). In addition, we used the synaptic marker cysteine string protein (CSP), a presynaptic vesicular protein [24]. We found that the HRP-labelled debris also colocalized with CSP (Fig. 2 G-I). Finally, we labeled the synapse against the postsynaptic glutamate receptor subunit D-GluRIIC antibody (Fig. 2 J-L) but we did not observe any debris immunoreactivity colocalizing with signal from this antibody. The presence of presynaptically driven GFP and CSP and the absence of the glutamate receptor in the HRP-labeled debris further validates the idea that these puncta are presynaptically derived.

Taken together, these results suggest that three main processes take place during muscle 4 NMJ dismantling. First, the postsynaptic components, in particular the DLG expressing endoplasmic reticulum surrounding the synaptic boutons initiates the dismantling process at 0 h APF. Secondly, a significant production of presynaptic debris shedding takes place by 2 h APF. Finally by 6 h APF the synaptic boutons appeared as a continuous structure. The motor axon decreases in size but never displays membrane breaks, which suggests that a motor neuron retraction mechanism occurs along with a massive disorganization of the postsynapse.

### Continuous Presence of Tubulin and Futsch Associated with the Loss of β-Spectrin and the Disorganization of FASII Indicates Motor Neuron Retraction

**Figure 3:**
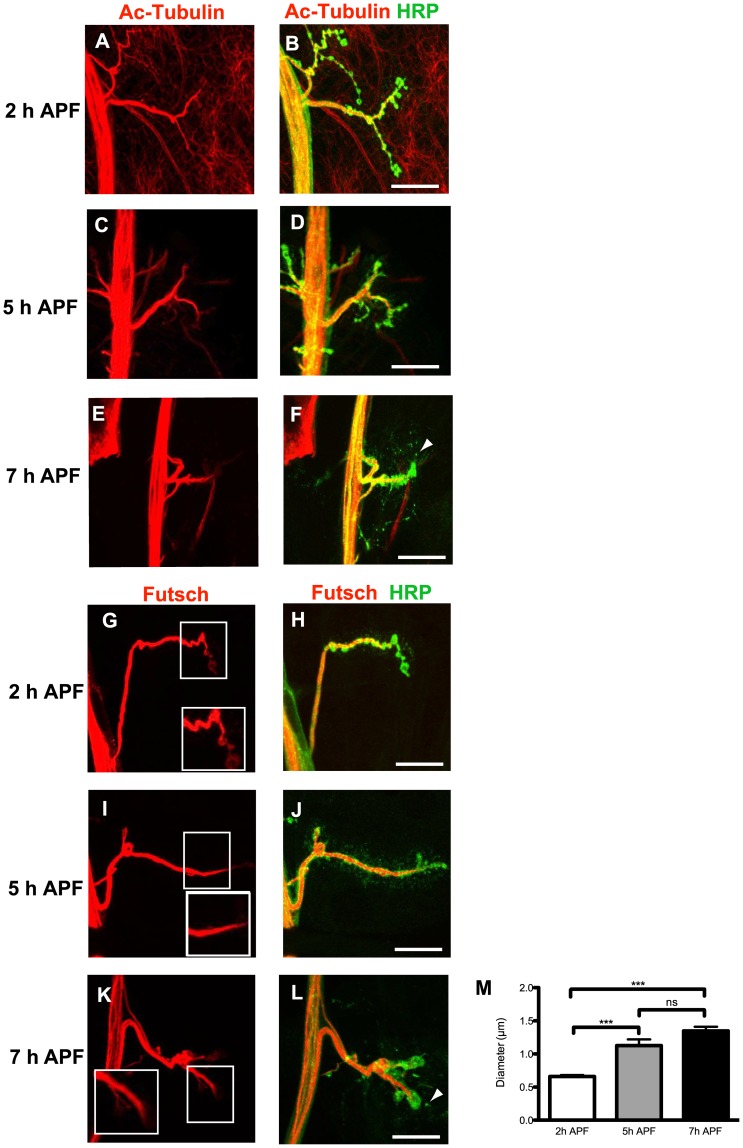
Microtubule disorganization and retraction but not degeneration take place during NMJ remodeling. A-F , Anti-Acetylated Tubulin (red) and anti-HRP (green) staining at 2 h, 5 h and 7 h APF. Tubulin staining was present and continuous at the NMJ in all the stages analyzed, and was absent in the HRP-labeled debris (arrowhead in F). **G-L**, Anti-Futsch (red) and anti-HRP (green) staining. The insets correspond to high magnifications. (I, J) 5 h APF, the presynaptic microtubules become thicker. (K, L) 7 h APF, the thickness of the microtubule bundle is maintained. Futsch was absent in HRP-labelled debris (arrowhead in L). Bars, 20 µm. **M**, Diameter of the distal end of the axons at 2 h (n = 17), 5 h (n = 16) and 7 h (n = 18) APF. Results are mean and s.e.m. ***: P<0.001, ns: no statistical difference (t-test). Genotype: *MHC-mGFP-Shaker.*

Even though membrane continuity was observed in the parent motor neuron, the presence of shed presynaptic debris does not alone allow us to completely discriminate between axonal degeneration or retraction. Tubulin and Futsch (the Map1b-like protein) are early molecular markers of degeneration. Thus, it is accepted that neuronal pruning associated with early Tubulin loss or with Tubulin bundle disruption reflects degeneration rather than retraction [2], [4], [25]. We first checked for the expression of Tubulin at 2 h, 5 h and 7 h APF. Acetylated Tubulin was present at all three stages as a continuous bundle (Fig. 3 A-F, and Fig. S3 A-F for higher magnifications and lower exposures). Continuous Tubulin staining was also observed at 10 h and 12 h APF late stages (Fig. S3 G-J). Similar staining was observed when we used a regular anti-Tubulin antibody (not shown). In addition, a continuous staining of microtubule bundles was observed at 2 h, 5 h and 7 h APF with an anti-Futsch antibody (Fig. 3 G-L). Interestingly, we noticed that the microtubule bundle becomes thicker between 2 h and 5 h APF and often does not extend to the distal end of the motor axon at 5 h APF (Fig. 3 M). Importantly, Tubulin and Futsch were absent from the presynaptic debris (Figure 3 F, L). These results further support our conclusion that motor neuron pruning occurs by axonal retraction along with debris shedding and not by axonal degeneration.

**Figure 4:**
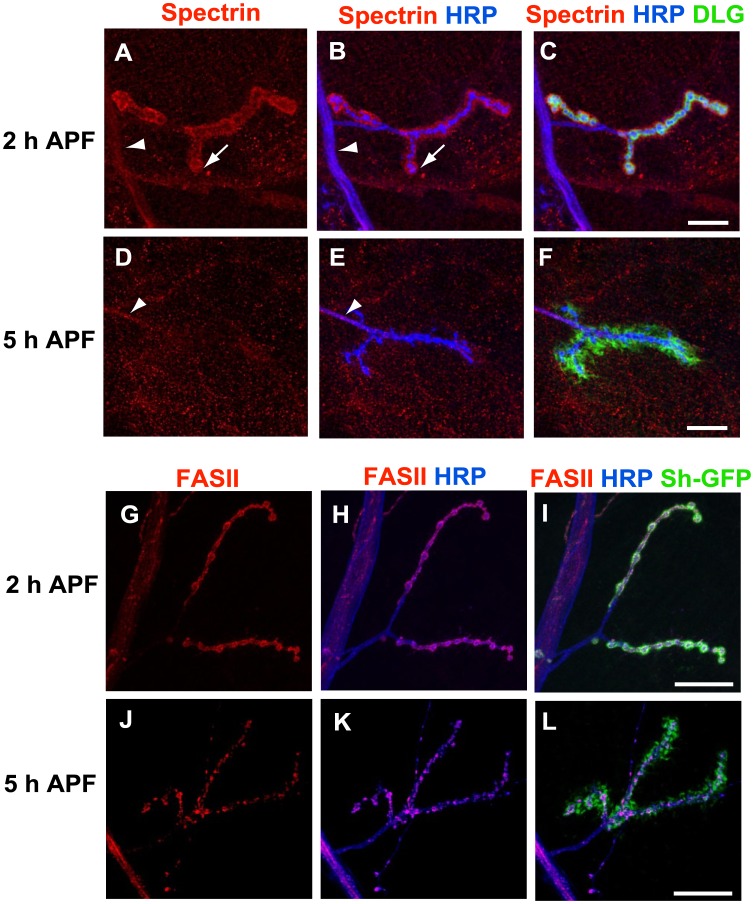
NMJ destabilization is correlated with the loss of β-Spectrin and the disorganization of the cell adhesion molecule FASII. A-F, anti-β-Spectrin (red), anti-HRP (blue) and anti-DLG (green). (A-C) 2 h APF β-Spectrin was detected in the motor nerve (arrowhead in A and B), in the synapse (arrow in A and B) and in the muscle (see also the co-localization with DLG as yellow in C). (D-F) β-Spectrin is lost in 5 h APF NMJ. Only traces were observed in axons (arrowhead in D and E). Bars, 20 µm. G-L, anti-FASII (red), anti-HRP (blue) and muscle-driven GFP by *MHC-mGFP-Shaker* (green). (G-I) The anti-FASII, anti-HRP and GFP co-localized throughout the NMJ 2 h APF. (J-L) The FASII staining was disorganized 5 h APF. The co-localization between FASII and HRP at the synapse is lost in K. Bars, 40 µm. Genotypes : (A-F) *y w^67c23^*, (G-L) *MHC-mGFP-Shaker.*

To better characterize the retraction mechanism we investigated the distribution of β-Spectrin because it is known that loss of this protein induces retraction in larval stages [26]. At 2 h APF, we found β-Spectrin in the motor nerve, the motor neuron axon and most probably into the presynaptic boutons (Fig. 4 A-C). This β-Spectrin pattern is similar to that reported in larval stages [26]. In addition, β-Spectrin was observed throughout the muscle and concentrated in the postsynaptic membranes surrounding the NMJ (Fig. 4 A-C). Conversely, β-Spectrin staining was absent at the synapse 5 h APF (Fig. 4 D-F) and only some traces were visible in the motor axon at this stage. Interestingly, overexpression of β-Spectrin in motor neurons (n = 14) with the *OK6-GAL4* driver did not block motor neuron retraction at 7 h APF. In this case, only 4% of presynapses showing arrested dismantling were observed, suggesting that β-Spectrin loss in motor neurons is not the unique early event leading to motor neuron retraction. Loss of presynaptic β-Spectrin has been correlated with an altered organization of FASII in larval motor neurons occurring before membrane retraction [26]. We observed that FASII was evenly distributed throughout the NMJ at 2 h APF (Fig. 4 G-I). Moreover, FASII co-localized with the muscle-driven GFP *MHC-mGFP-Shaker* at this stage (Fig. 4 I). In contrast, at 5 h APF, FASII expression was completely disorganized (Fig. 4 J-L). There were regions in which HRP was present but the FASII staining was disrupted. Thus, the loss of β-Spectrin and Fasciclin appear to clearly mark the early events in the destabilization of the NMJ prior to motor neuron retraction.

### Glia Invade NMJ Early at Metamorphosis, Dynamically Retract before Motor Neurons and May be Necessary for NMJ Dismantling

**Figure 5:**
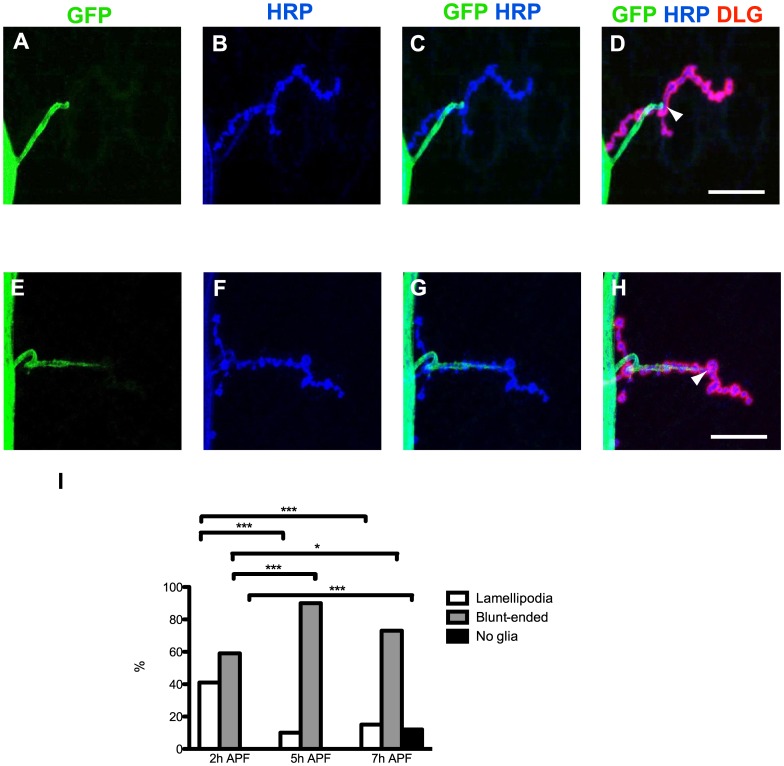
Glia invade NMJ early in metamorphosis and dynamically retracts before motoneuron pruning. A-H, Glial extensions were observed (green) at 2 h APF by expressing *UAS-mCD8-GFP* with the *repo-GAL4* driver. The NMJ was also labeled with HRP (blue) and DLG (red). (A-D) A blunt-ended glial process is seen in A. A lamellipodium process is shown in E. Additional merges including the anti-DLG staining are shown in D and H. Here, the arrowheads point to the end of the glial extension. Bars, 30 µm. **I**, Glial projections were scored at 2 h (n = 17), 5 h (n = 20) and 7 h (n = 26) APF. The percentages of lamellipodia, blunt-ended and no glial extensions are represented. *: P<0.05, ***: P<0.001 (χ^2^ test). Genotype: *2× UAS-mGFP/+*; *repo-GAL4/+.*

In the fly, glia play an essential role in axonal pruning [27], [28], [29], [30]. We checked for the presence of glial extensions at the NMJ during metamorphosis (Fig. 5). For that purpose, we expressed two copies of a membrane tethered GFP (*mCD8-GFP)* in glia, using either *repo-GAL4* or *Gliotactin-GAL4,* and identified NMJs by HRP-labeling. At 2 h APF, 41% of the NMJ analyzed showed lamelipodia-like glial extensions spreading further out the axon branch point and enveloping several boutons (Fig. 5 I), while 59% of the NMJ analyzed terminated exactly at the axon branch point before the beginning of synaptic boutons (blunt-end). At 5 h APF, we observed only 10% of the NMJ contacted by lamelipodia and 90% by blunt-end glia. Similar results were observed with *Gliotactin-GAL4* driving *mCD8-GFP* (not shown). These observations suggest that the glial extensions likely retract 5 h APF, just before motor neuron retraction is observed.

**Figure 6:**
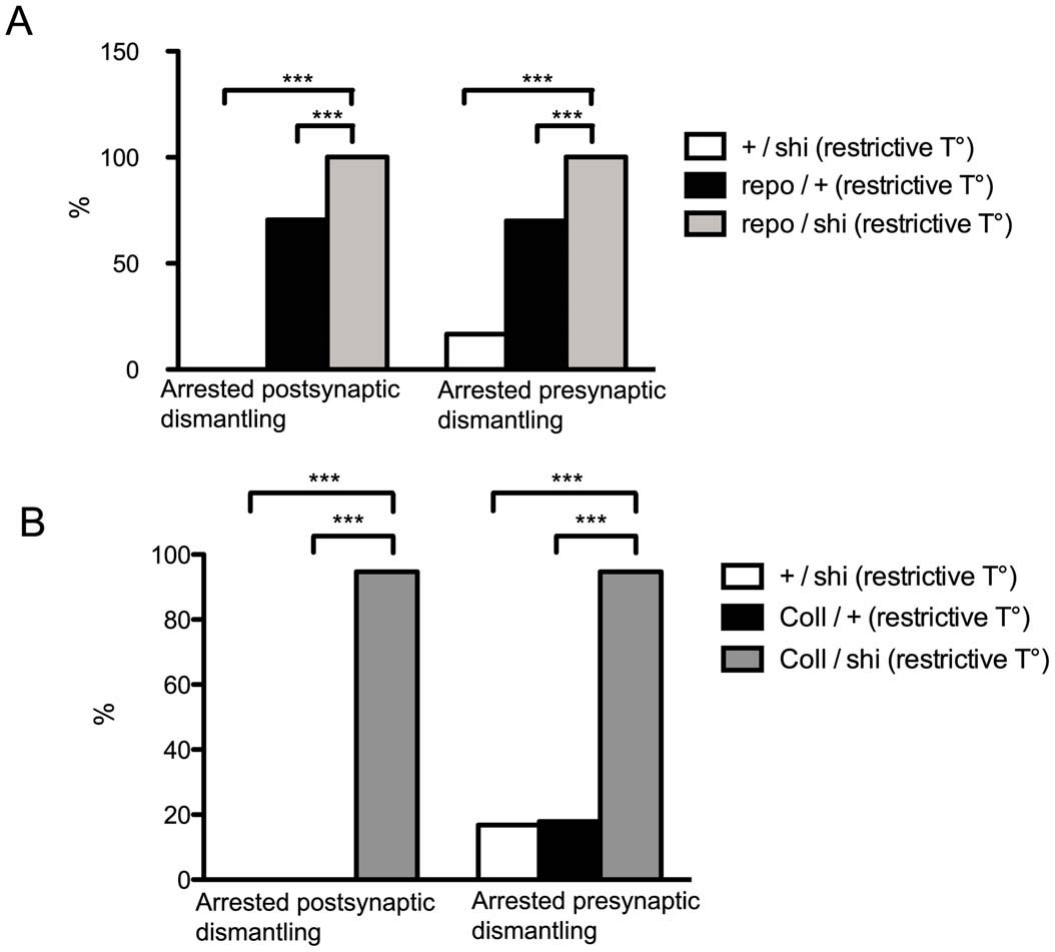
Disrupting glial and phagocyte function blocks NMJ dismantling. Percentage of 7 h APF NMJ showing arrested dismantling at restrictive temperature in **A**: +/*shi* (n = 36), *repo*/+ (n = 95) and *repo/shi* (n = 40). **B**: +/*shi* (n = 36), *Coll*/+ (n = 28) and *Coll/shi* (n = 57). Expression of *shi^ts^* in glia and in phagocytes significantly increased the percentage of arrested post and presynaptic dismantling phenotypes. ***: P<0.001 (χ^2^ test). Genotypes: A: +/shi  =  +/*UAS-shi^ts1^*. repo/+  =  *repo-GAL4/+.* repo/shi  =  *repo-GAL4/UAS-shi^ts1^* B: +/shi  =  +/*UAS-shi^ts1^.* Coll/+  =  *Collagen-GAL4*/+. Coll/shi  =  *Coll-GAL4*/*UAS-shi^ts1^.*

The *shibire* (*shi*) gene encodes for dynamin guanosine triphosphatase, which is involved in membrane related functions such as phagocytosis and endo-exocytosis. Targeted expression of *shi^ts1^* inhibits these membrane functions because the protein produced by the temperature-sensitive allele *shi^ts1^* acts as a dominant-negative at a restrictive temperature [21]. We disrupted glial function by over-expressing the *UAS-shi^ts1^* specifically in glial cells using the *repo-GAL4* driver and analyzed NMJs (Fig. 6 A). Under these conditions we observed arrested pre and postsynaptic dismantling phenotypes in 100% of the samples at 7 h APF. 0% and 70% of arrested postsynaptic dismantling as well as 17% and 70% of arrested presynaptic dismantling were obtained in *+/UAS-shi^ts1^* and *repo-GAL4/+* controls, respectively. There is a nearly 50% increase in the mutant phenotype when *shi^ts1^* is associated to *repo-GAL4*. Nevertheless, since there is a clear dominant effect of the *repo-GAL4* alone, it is difficult to definitively conclude to which extent glia participates in NMJ dismantling. However, we reasoned that if glia retraction occurring 5 h APF destabilizes NMJ, thus, mutant NMJ displaying arrested post and presynaptic dismantling at 7 h APF might, contrary, present glial extensions (as lamelipodia) leading to NMJ stabilization. Consequently, we expressed *UAS-shi^ts1^* in glial cells using a *repo-GAL4, UAS-GFP* line, in which glial cells are GFP-labeled, and quantified glial extensions at restricted temperature. We also labeled NMJ with HRP and DLG to visualize the pre and the postsynapse. Under these conditions, as expected, we observed 100% of postsynapses displaying arrested dismantling and 100% of presynapses showing arrested dismantling (n = 11). Here, 72% of NMJ displayed glial extensions at 7 h APF. In controls *repo-GAL4, UAS-GFP/+*, 50% of NMJ showed arrested dismantling (n = 14), all of them presenting glial extensions. The 50% of dismantled NMJ did not display any glial extensions (0%), suggesting a direct link between the presence of glial extensions and the maintenance of larval NMJ.

### Disruption of Phagocytes Function Blocks Larval NMJ Dismantling

At metamorphosis, phagocytic blood cells appear to be closely involved in dendrite pruning of da neuron dendrites [4]. Accordingly, we asked if phagocytic blood cells were involved in NMJ dismantling. Therefore, we expressed *UAS-shi^ts1^* specifically in phagocytes using the *Collagen-GAL4* (*Coll-GAL4*) driver [31]. This expression at restrictive temperature resulted in 95% of postsynapses and 95% of presynapses in which dismantling was arrested at 7 h APF (Fig. 6 B). 0% and 0% of postsynapses as well as 17% and 18% of motor neurons presented arrested dismantling in *+/UAS-shi^ts1^* and *Coll-GAL4/+* controls, respectively. In addition, we used the *Hemolectin-GAL4* (*Hml-GAL4*) [14] and the *Peroxidasin-GAL4 (Pxn-GAL4)* [16] phagocyte-specific lines to express *UAS-shi^ts1^* at restrictive temperature in those cells. Expression of *UAS-shi^ts1^* with both *GAL4* lines resulted in 100% of postsynapses and 100% motor neurons displaying arrested dismantling phenotypes at 7 h APF (Fig. S4). 33% and 7% of postsynapses as well as 33% and 7% of presynapses showing arrested dismantling were obtained in *Hml-GAL4/+* and *Pxn-GAL4/+* controls, respectively. The strong increase in blocking the NMJ phenotypes suggests a crucial role for phagocytes in NMJ dismantling.

### The Nuclear Receptor Pathway Regulates *EcR-B1* Expression in both Muscles and Motor Neurons

During metamorphosis, the steroid ecdysone receptor *EcR-B1* is responsible for neuron remodeling in a cell autonomous way. It was recently reported that *EcR-B1* is involved in NMJ postsynaptic dismantling and likely in motor neuron pruning also [9]. We have confirmed that *EcR-B1* plays key functions not only in postsynaptic dismantling but also in motor neuron retraction and that muscle dismantling is instructive on motor neuron retraction (result not shown). Then, the crucial question now is to determine the *EcR-B1* regulatory pathways leading to ECR-B1 expression in the pre and postsynaptic compartments.

**Figure 7:**
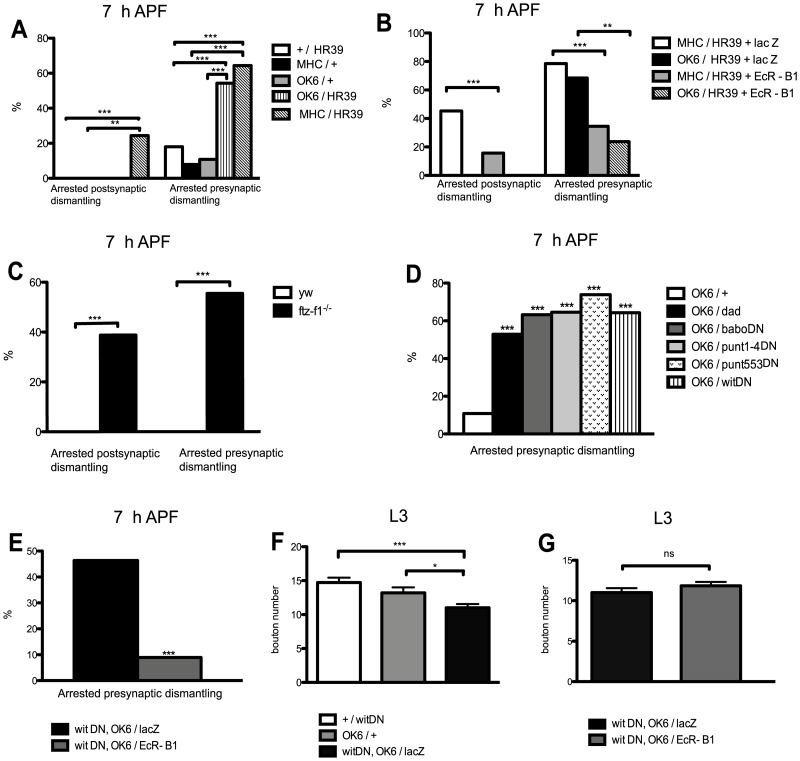
The nuclear receptor pathway regulates *EcR-B1* expression in both muscles and motor neurons but the TGF-β pathway regulates *EcR-B1* expression only in motor neurons. **A**, Percentages of 7 h APF NMJ showing arrested post and presynaptic dismantling in: +/*Hr39* (n = 56), *MHC*/+ (n = 38), *OK6*/+ (n = 37), *OK6/Hr39* (n = 35) and *MHC/Hr39* (n = 45) pupae. **B**, Percentages of 7 h APF NMJ showing arrested post and presynaptic dismantling in: *MHC/Hr39+ lacZ* (n = 42), *OK6/Hr39+ lacZ* (n = 19), *MHC/Hr39+ EcR-B1* (n = 127) and *OK6/Hr39+ EcR-B1* (n = 32) pupae. **C**, Percentages of 7 h APF NMJ showing arrested post and presynaptic dismantling phenotypes in: *ftz-f1^−/−^* (n = 36) and *yw* (n = 18) pupae. **D**, Percentages of 7 h APF NMJ showing arrested post and presynaptic dismantling in different TGF-β signaling mutant backgrounds: *OK6*/+ controls (n = 37), *OK6/dad* (n = 17), *OK6/babo^DN^* (n = 19), *OK6/punt^1-4DN^* (n = 31) and *OK6/punt^553DN^* (n = 23), *OK6/wit^DN^* (n = 14). **E**, The arrested presynaptic dismantling phenotype obtained in *wit^DN^, OK6/lacZ* individuals (n = 41) was rescued in *wit^DN^, OK6/EcRB1* in 7 h APF motor neurons (n = 56). **F**, At larval stages, bouton number in *wit^DN^, OK6/lacZ* individuals (n = 18) was lower than in +/*wit^DN^* (n = 18) and in *OK6*/+ (n = 20). **G**, At larval stages, the bouton number in *wit^DN^, OK6/lacZ* individuals (n = 18) was similar than in *wit^DN^, OK6/EcR-B1* (n = 19). Results are means and s.e.m. The differences are significant in a χ^2^ test (**: P value is <0.01, ***: P<0.001) or not statistically different (ns) for A-E. The differences are significant in a t-test (*: the two-tailed P value is <0.05, ***: P<0.001) or not statistically different (ns) for F and G. Genotypes: A: +/HR39 =  +/*UAS-Hr39*. MHC/+  =  *MHC-GAL4/+.* OK6/+  =  *OK6-GAL4/+.* OK6/HR39 =  *OK6-GAL4/UAS-Hr39.* MHC/HR39 =  +/*UAS-Hr39; MHC-GAL4/+.* B: MHC/HR39+ lacZ * =  +/UAS-Hr39, UAS-lacZ; MHC-GAL4/+.* OK6/HR39+ lacZ * =  OK6-GAL4/UAS-Hr39, UAS-lacZ.* MHC/HR39+ EcR-B1* =  MHC-GAL4/UAS-Hr39, UAS-EcR-B1.* OK6/HR39+ EcR-B1* = OK6-GAL4/+; UAS-Hr39, UAS-EcR-B1/+.* C: yw  =  *y w^67c23^*. ftz-f1^−/−^  =  *ftz-f1^17^*/*ftz-f1^19^*. D: OK6/+  =  *OK6-GAL4/+*. OK6/dad  =  *OK6-GAL4/UAS-dad.* OK6/baboDN  =  *OK6-GAL4/2xUAS-baboΔI*; +/2x*UAS-baboΔI*. OK6/punt1-4DN  =  *OK6-GAL4/2xUAS-puntΔI.* OK6/punt553DN  =  *OK6-GAL4/2xUAS-puntΔI.* OK6/witDN  =  *OK6-GAL4/UAS-wit^DN^*. E and G : witDN, OK6/lacZ  =  *UAS-wit^DN^, OK6*-*GAL4*/+; *UAS-lacZ/*+. witDN, OK6/EcR-B1 =  *UAS-wit^DN^*, *OK6-GAL4/+; UAS-EcR-B1/+.* (F): +/witDN  =  +/*UAS-wit^DN^*. OK6/+  =  *OK6-GAL4/+*. witDN, OK6/lacZ  =  *UAS-wit^DN^, OK6*-*GAL4*/+; +/*UAS-lacZ*.

In order to test if the nuclear receptor pathway was involved in NMJ dismantling, we first over-expressed *UAS-Hr39* specifically in muscles with the *MHC-GAL4* driver (Fig. 7 A). More than 20% of postsynapses showed arrested dismantling in *MHC/Hr39* individuals at 7 h APF. No postsynapses showing arrested dismantling (0%) were observed in +/*Hr39* and *MHC*/+ controls. In addition, this arrested postsynaptic dismantling phenotype was correlated with an arrested presynaptic dismantling phenotype (64.4% in individuals over-expressing *Hr39* in muscles compared to 18% and 7.9% in +/*Hr39* and *MHC*/+ controls respectively). Next, we asked if the nuclear receptor pathway may also have a direct role in motor neuron pruning. Consequently, we over-expressed the *UAS-Hr39* specifically in motor neurons with the *OK6-GAL4* driver (Fig. 7 A). A high percentage of arrested motor neuron dismantling phenotypes (54%) was scored in *OK6/Hr39* individuals compared to controls (18% in +/*Hr39* and 10.8% in *OK6*/+). In contrast, an absence of arrested postsynaptic dismantling phenotypes was observed in *OK6/Hr39* individuals. Taken together, these results suggest that the nuclear receptor pathway is needed in both muscles and motor neurons for NMJ remodeling.

**Figure 8:**
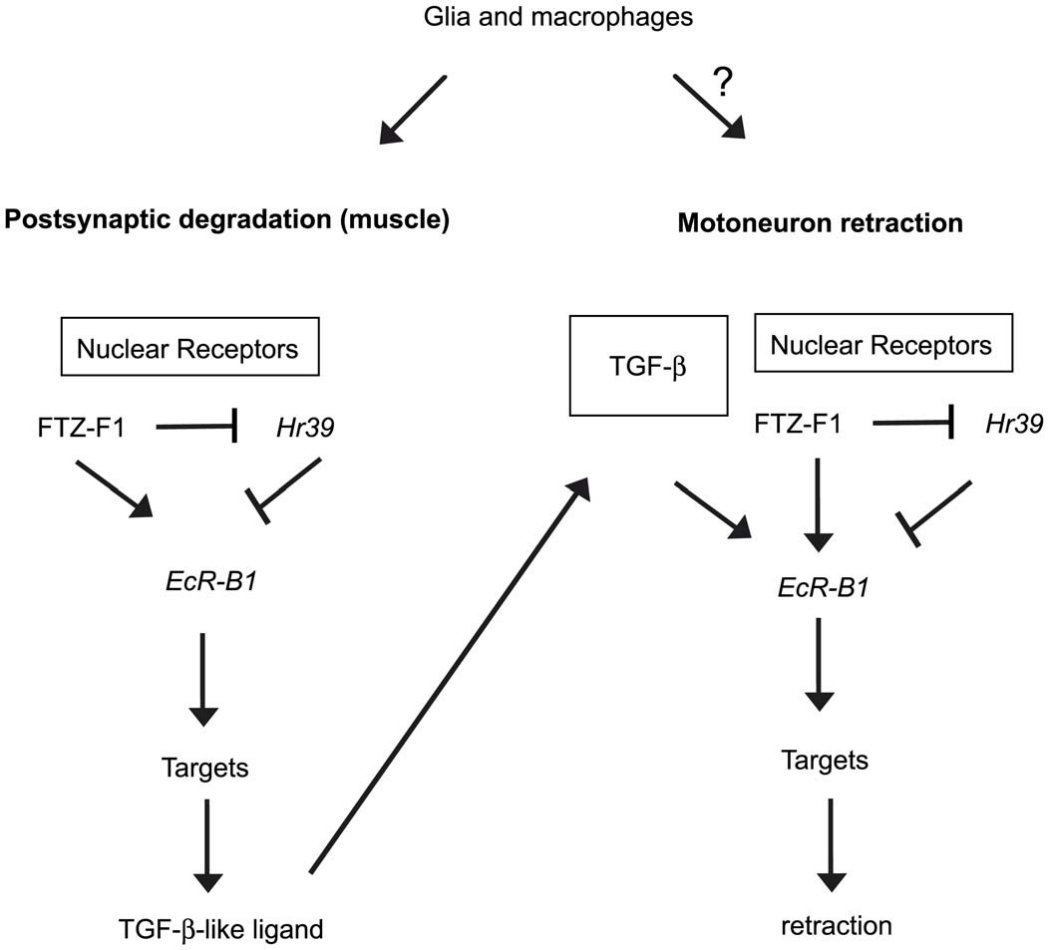
NMJ dismantling model. The nuclear receptor pathway involving FTZ-F1 and HR39 regulates the *EcR-B1* expression in muscle and initiate dismantling. FTZ-F1 and HR39 positively and negatively respectively control the *EcR-B1* expression at the postsynapse. FTZ-F1-mediated *EcR-B1* expression leads to transcriptional regulation of target genes involved in postsynaptic degradation. Then, TGF-β/BMP signaling is supposed to be induced in response to postsynaptic degradation. We propose that this signal produced by the dismantling muscle is received by the motor neuron where activation of *EcR-B1* expression (by TGF-β signaling and nuclear receptor) triggers the axon retraction. In addition, glial cells and macrophages participate to the NMJ dismantling process.

If we assume that HR39 negatively regulates *EcR-B1* then expression of both *UAS-Hr39* and *UAS-EcR-B1* should lead to rescue NMJ remodeling. To test this hypothesis, we over-expressed both *UAS-Hr39* and *UAS-EcR-B1* either in muscles or motor neurons and compared the percentage of pre and postsynapses presenting arrested dismantling at 7 h APF to control individuals co-expressing both *UAS-Hr39* and *UAS-lacZ* (Fig. 7 B). We controlled for the possibility that co-expression of *UAS-EcR-B1* diluted the amount of available GAL4, thereby decreasing the efficiency of *UAS-Hr39*, by co-expressing a neutral transgene, *UAS-lacZ*, along with *UAS-Hr39*. Interestingly, double expression of *Hr39* and *EcR-B1* in muscles rescued both the arrested postsynaptic dismantling (15% in *Hr39+ EcR-B1* individuals compared to 45% in *Hr39+ lacZ* individuals) as well as the arrested presynaptic dismantling phenotypes (34% in *Hr39+ EcR-B1* individuals compared to 79% in *Hr39+ lacZ* individuals). In addition, co-expression of *UAS*-*Hr39* and *UAS-EcR-B1* in motor neurons rescued the arrested presynaptic dismantling phenotype (24% of arrested presynaptic dismantling in *Hr39+ EcR-B1* individuals compared to 69% of arrested presynaptic dismantling in *Hr39*+ *lacZ* individuals).

*ftz-f1* is required for muscle-driven morphogenetic events at the prepupal-pupal transition in *Drosophila* [32]. Therefore, it is likely that *ftz-f1* is expressed in muscles during NMJ metamorphosis. We used the allelic combination *ftz-f1^17^*/*ftz-f1^19^* (viable at pupal stages) to reduce the endogenous *ftz-f1* function [32]. We observed a high percentage of both arrested postsynaptic (38%) and presynaptic (55.5%) dismantling in these individuals compared to controls (0% showing arrested postsynaptic dismantling and 0% showing arrested presynaptic dismantling) (Fig. 7 C). These results strongly suggest a role for *ftz-f1^+^* in NMJ remodeling.

### The TGF-β/BMP Pathway Regulates the *EcR-B1* Expression in Motor Neurons

It has been shown that the TGF-β/Activin pathway mediated by the type I receptor *babo* and the type II receptors *wit* and *punt* activates *EcR-B1* expression in mushroom body γ neurons which in turn leads to the degeneration of γ axons [5]. We first tested if TGF-β/Activin signaling reception acts presynaptically in NMJ dismantling. Consequently, different dominant negative forms of TGF-β receptors were expressed in motor neurons in order to block the receptor functions. Expression of *UAS-babo^DN^*, *UAS-punt^1-4DN^*, *UAS-punt^553DN^*, and *UAS-wit^DN^* with the *OK6-GAL4* driver led to 63%, 65%, 74% and 64% of arrested presynaptic dismantling phenotypes respectively at 7 h APF, significantly higher than the 11% of arrested presynaptic dismantling phenotypes observed in *OK6-GAL4*/+ controls (Fig. 7 D). Similarly, no significant effects above this control value were observed in *UAS-babo^DN^* (15%, n = 27), *UAS-punt^553DN^* (5%, n = 39), and *UAS-wit^DN^* (15%, n = 27) individuals without a GAL4 driver. We also over-expressed *UAS-dad* in motor neurons. *dad* has been shown to specifically inhibit *Mad*-mediated signaling which results from phosphorylation via the BMP side of the pathway [33]. Interestingly, over-expression of *UAS-dad* also led to 53% of arrested presynaptic dismantling phenotypes (Fig. 7 D) although the *UAS-dad/+* control led to ∼10% (n = 43), not statistically significant compared to the +/+ control. Thus, it is possible that both *Mad*-dependent BMP signaling and TGF-β/Activin signaling are essential for motor neuron pruning either independently or as a result of pathway crosstalk due to mixed R-SMAD complexes [34] whose stoechiometry could have been disrupted by DAD over-expression.

We also expressed the dominant negative receptors and DAD specifically in muscles and did not observe any significant changes in the percentage of postsynaptic remodeling at 5 h APF (0% of arrested postsynaptic dismantling in: *MHC/dad* n = 19, *MHC/babo^DN^* n = 27, *MHC/punt1-4^DN^* n = 3, *MHC/wit^DN^* n = 37) compared to controls (0% of arrested postsynaptic dismantling in *MHC/+* n = 24). As expected, the expression of the different dominant negative receptors or DAD in muscles at 5 h APF do not affect the percentage of presynaptic dismantling (*MHC/babo^DN^:* 100%, n = 27; *MHC/punt1-4^DN^:* 100%, n = 3; *MHC/wit^DN^:* 100%, n = 37; *MHC/dad:* 100%, n = 19) compared to controls (*MHC/+:* 100%, n = 24). Similarly, we did not observe any significant changes in the percentage of postsynaptic remodeling at 7 h APF (*MHC/wit^DN^ :* 3%, n = 31) compared to controls (*MHC/+:* 8%, n = 24). Furthermore, several TGF-β receptor RNAi constructs were driven in muscles using a *MHC-GAL4* line expressing *UAS-Dicer2*, at 29°C to improve the RNAi action [35]. As expected based on the dominant negative data, expression of *UAS-babo RNAi* (n = 10), *punt RNAi (n = 4)* and *Mad RNAi* (n = 4) in muscle did not block postsynaptic remodeling compared to controls (*UAS-Dicer2/+*; *MHC-GAL4*/+: 0% of arrested pre and postsynaptic remodeling).

Furthermore, we tested if the TGF-β pathway could regulate *EcR-B1* expression during motor neuron pruning and compared the percentage of arrested presynaptic dismantling phenotypes of individuals expressing *UAS-wit^DN^* as well as an *UAS-lacZ* in order to rule out potential GAL4 titration, driven by the *OK6-GAL4* driver with individuals expressing *UAS-wit^DN^* as well as *UAS-EcR-B1* (Fig. 7 E). The 46% of presynapses displaying arrested dismantling obtained in *wit^DN^, OK6/lacZ* individuals was rescued to 9% in *wit^DN^, OK6/EcR-B1* individuals. These results strongly suggest that the TGF-β/BMP pathway also regulates the *EcR-B1* expression during motor neuron retraction. Thus, in contrast to what we observed for the nuclear receptor pathway, which is essential in both muscle and motor neurons during NMJ remodeling, the TGF-β/BMP signaling must only be active in the motor neurons for proper NMJ remodeling.

To determine if *EcR-B1* was a direct target of the TGF-β pathway we analyzed the expression of ECR-B1 in late larval motor neurons under two different TGF-β pathway mutant conditions. First, we quantified ECR-B1 expression in *wit* mutants (Fig. S5 A-C, G). For this we used the allelic combination *wit^A12^/wit^B11^* (viable at late larval stages) to reduce the endogenous *wit* function [15]. We choose lower regions of the ventral cord and focused on the intermediate and superficial part, which contains U1 to U5 motor neurons projecting to abdominal muscles [36]. U1 to U5 motor neurons were labeled with antibodies against EVE. Under these conditions, we observed a statistically significant reduction of the ECR-B1 expression levels in the EVE-positive motor neurons (402±105.2; n = 25 quantifications in 5 different nerve cords) compared to controls *yw* (598±147.4; n = 25 quantifications in 5 different nerve cords) (Fig. S5 G). Second, we used the *OK6-GAL4* driver to express both *UAS-wit^DN^* and *UAS-mCD8-GFP* in motor neurons (Fig. 5S D-F, H). This line allowed us to visualize GFP-labeled motor neurons. Then, we quantified the ECR-B1 expression levels in GFP-positive motor neurons and selected the U1–U5 motor neurons. Under these conditions expression of ECR-B1 was lower (431.44±145.62; n = 25 quantifications in 5 different nerve cords) than in controls *OK6-GAL4/UAS-GFP* (1293.35±385.4; n = 25 quantifications in 5 different nerve cords) (Fig. S5 H). As an additional control, we quantified the ECR-B1 expression in cells GFP negative in mutants and controls and we did not observe any statistically significant difference (*UAS-wit^DN^, OK6-GAL4/2xUAS-GFP*  = 575.8±130.7, n = 25 and *OK6-GAL4/2xUAS-GFP*  = 499.9±137.9, n = 25. P = 0.0517).

### The TGF-β Pathway Mediates Independent Regulation of Motor Neuron Retraction and NMJ Growth

TGF-β/BMP signaling mediated by the *wit* receptor was originally characterized at the *Drosophila* NMJ as being required for normal NMJ growth [12], [15]. Consequently we counted the muscle 4 bouton number (I_b_ boutons) at the abdominal segment 3 in third instar larvae expressing *UAS-wit^DN^* in motor neurons with the *OK6-GAL4* driver and compared to the bouton number present in larvae expressing either *UAS-wit^DN^* or *OK6-GAL4* alone as a control (Fig. 7 F). The number of synaptic boutons was slightly but significantly reduced when the *UAS-wit^DN^* was driven in motor neurons (11±0.56) compared to *UAS-wit^DN^*/+ or *OK6-GAL4*/+ controls (14.72±0.72 and 13.2±0.78 respectively). To determine whether the blockade of motor neuron pruning observed in pupae expressing *UAS-wit^DN^* in motor neurons may be the result of impaired synaptic growth we quantified the I_b_ bouton number in larvae co-expressing *UAS-wit^DN^* and *UAS-lacZ* (11±0.56) and larvae co-expressing *UAS-wit^DN^* and *UAS-EcR-B1* (11.84±0.49) in motor neurons under the control of the *OK6-GAL4* driver. The difference in the bouton number was not statistically significant (Fig. 7 G). Consequently, since the synaptic bouton number was similar at L3 but the motor neuron pruning phenotype was modified, we conclude that motor neuron retraction phenotype is not a secondary consequence of impaired synaptic growth.

## Discussion

It is a general feature of maturing brains, both in vertebrates and in invertebrates, that neural circuits are remodeled as the brain acquires new functions [1]. In holometabolous insects, the difference in lifestyle is particularly apparent between the larval and the adult stages. These insects possess two distinct nervous systems at the larval and adult stages. A class of neurons is likely to function in both the larval and the adult nervous systems. The neuronal remodeling occurring during this developmental period is expected to be necessary for the normal functioning of the new circuits.

### Axon Retraction versus Degeneration

The pruning of an axon can involve a retraction of the axonal process, its degeneration or both a retraction and degeneration. The MB γ axon is pruned through a local degeneration mechanism [2]. On the other hand, axons may retract their cellular processes from distal to proximal in the absence of fragmentation and this mechanism is called retraction. Interestingly, the two mechanisms can occur sequentially in the same neuron, as in the case of the dendrites of the da neurons, where branches degenerate and the remnant distal tips retract [4].

Here, we provide evidence that the motor neuron innervating larval muscle 4 (NMJ 4) is pruned predominantly through a retraction mechanism. The first morphological indication of motor neuron retraction is the absence of fragmentation observed with anti-HRP staining at the level of the presynapse in all the developmental stages analyzed, together with a decrease in perimeter size observed after 2 h APF. The continuity of this HRP staining is in contrast to the pronounced interruptions between blebs observed with an antibody against mCD8 in γ axons [2] and also with the mCD8-GFP labeled fragments observed during da dendrites pruning [4]. A molecular indication of motor neuron retraction in our studies is the fact that β-Spectrin disappears at the synapse 5 h APF, before motor neuron pruning takes place. Indeed, it has been shown using an RNA interference approach [26] that loss of presynaptic β-Spectrin leads to presynaptic retraction and synapse elimination at the NMJ during larval stages. The modifications of the microtubule morphology that we have observed, such as an increase in microtubule thickness and withdrawal, provide additional evidence of axonal retraction during NMJ remodeling. Finally, a strong argument in favor of a motor neuron retraction mechanism is the fact that Tubulin is present at the NMJ throughout all stages of axonal pruning at the start of metamorphosis (0–7 h APF). This stands in clear contrast to the abolition of Tubulin expression observed before the first signs of γ axon degeneration [2]. It is also interesting to note that the motor neuron retraction observed here at metamorphosis and at larval stages [26] are morphologically different. During metamorphosis, we never visualized retraction bulbs or postsynaptic footprints, which have been reported at larval stages. The fact that the postsynapse dismantles at metamorphosis before motor neuron retraction might explain these discrepancies. Worth noting is the mechanistic correlation between accelerated debris shedding observed here for NMJ pruning at the start of metamorphosis and axosome shedding occurring during vertebrate motor neuron retraction [37].

### Role of Glial Cells and Phagocytes

In vertebrates, glia play an essential role in the developmental elimination of motor neurons [37]. In *Drosophila*, the role of glia in sculpting the developing nervous system is becoming more apparent. Clear examples of a role for engulfing glial cells in axon pruning are well documented during the MB γ axon degeneration at metamorphosis [27], [28], [29], [30]. Also, glia are required for clearance of severed axons of the adult brain [38], [39]. A distinct protective role of glia has been recently discovered during the patterning of dorsal longitudinal muscles by motor neurobranches [40]. Here, we describe the presence of glia processes close to the end of the pupal NMJ. Our observations suggest that the glial extensions retract at 5 h APF, just before motor neuron retraction is observed. When the glial dynamic is blocked, the NMJ dismantling might be also blocked. We hypothesize that during development in larvae and early pupae, glial processes have a protective role and aid in the maintenance of the NMJ. Then, between 2 and 5 h APF, glial retraction would be a necessary initial step that allows NMJ dismantling. In accordance with this hypothesis, glia play a protective role in the maintenance of NMJ during pruning of second order motor neuron branches 31 h APF [40]. In contrast, our results are opposite to the work reported by Liu, Z. *et al* [9] in which neither glial extensions nor glial function in NMJ dismantling were identified. One possible explanation for the differences in our findings is that Liu, Z. *et al* analyzed the morphology and role of glia at 9 h APF, a time which is most likely too late. Indeed, we only observed 10% of NMJ displaying glial extensions at 7 h APF.

Disruption of *shi* function specifically in glial cells results in an unpruned mushroom body γ neuron phenotype and prevents glial cell infiltration into the mushroom body [27]. One can note that at the NMJ the role of the glia is proposed to be essentially opposite from its role in MB γ axons pruning but in both cases blocking the glia dynamics results in a similar blocking of the pruning process.

In vertebrates, phagocytes are recruited to the injured nerve where they clear, by engulfment, degenerating axons [41], [42]. In *Drosophila*, phagocytic blood cells engulf neuronal debris during elimination of da sensory neurons [4]. We show here that blocking phagocyte dynamics with *shi* produces a strong blockade of the NMJ dismantling process. One possibility is that phagocytes attack and phagocytose the postsynaptic material, a process blocked by compromising *shi* function resulting in postsynaptic protection. In accordance, it has been shown that phagocytes attack not only the da dendrites to be pruned, but also the epidermal cells that are the substrate of these dendrites [4].

During NMJ dismantling, the muscle has an instructive role for motor neuron retraction. In all the situations where postsynapse dismantling is blocked, the corresponding presynaptic motor neuron retraction is also blocked (see below for a discussion on the molecular mechanism). Therefore, it is sufficient to propose that both glial cells and phagocytes affect only the postsynaptic compartment. Nevertheless, one cannot rule out that these two cell types both act directly at the pre and at the postsynapse.

### Sequential Activation of *EcR-B1*: the Nuclear Receptor Pathway in the Muscle May Induce TGF-β Signaling in Motor Neuron

ECR-B1 is highly expressed and/or required for pruning in remodeling neurons of the CNS [3], [43], [44], [45]. MB γ neurons and antennal lobe projection neurons remodeling require both the same TGF-β signaling to upregulate *EcR-B1* [5], [44]. In the MBs only neurons destined to remodel show an upregulation of *EcR-B1*. At least two independent pathways insure *EcR-B1* differential expression. The TGF-β pathway and the nuclear receptor pathway are thought to provide the necessary cell specificity of *EcR-B1* transcriptional activation [3], [6], [46]. We show here that in the motor neuron pruning these two pathways are also necessary to activate *EcR-B1*. Noteworthy, showing an analogous requirement of *ftz-f1/Hr39* pathway in two different remodeling neuronal systems unravels the fundamental importance of this newly described pathway.

Figure 8 presents the model we propose for the sequential events that are occurring during NMJ dismantling at early metamorphosis. First, *EcR-B1* is expressed in the muscle under the control of FTZ-F1. FTZ-F1 activates *EcR-B1* and represses *Hr39*. This repression is compulsory for *EcR-B1* activation. Importantly, TGF-β/BMP signaling does not appear to be required for *EcR-B1* activation in this tissue, however, a result of *EcR-B1* activation in the muscle would be the production of a secreted TGF-β family ligand. Then, this secreted TGF-β family ligand reaches the appropriate receptors and activates the TGF-β signaling in the motor neuron. Finally, TGF-β signaling in association with the nuclear receptor pathway activates *EcR-B1* expression resulting in motor neuron retraction. Since glial cells and phagocytes are required for the dismantling process, it is possible that a TGF-β/BMP family ligand(s) be produced by one or both of these cell types and not by the postsynaptic compartment. Noteworthy, a recent study shows that glia secrete myoglianin, a TGF-β ligand, to instruct developmental neural remodeling in *Drosophila* MBs [46]. Nevertheless, one can note that the requirement of the two pathways in the motor neuron provides a simple molecular explanation of the instructive role of postsynapse degradation on motor neuron retraction. This mechanism insures the temporality of the two processes and prevents motor neuron pruning before postsynaptic degradation. It was proposed that in the MBs, the association of these two pathways provides the cell (spatial) specificity of pruning. Here, this association is proposed to provide the temporal specificity of the events. Future studies will be necessary to understand how *EcR-B1* controls the production of a TGF-β/BMP ligand(s) in the muscle, the reception of this signal by the motor neuron and the ultimate response by the motor neuron to initiate retraction. These steps will be necessary to unravel the molecular mechanisms underlying the NMJ dismantling process and related phenomenon in vertebrate NMJ development and disease. Interestingly, it appears that TGF-β ligands on the one hand are positive regulators of synaptic growth during larval development and on the other hand, they are positive regulators of synaptic retraction, at the onset of metamorphosis. In both situations signaling provides a permissive role, sending a signal from the target tissue to the neuron. The consequence of this signal would be dependent on developmental timing thus, on a change in context.

## Supporting Information

Figure S1 **NMJ dismantling during** ***Drosophila*** **metamorphosis (continued).** Composite confocal images of the NMJ at muscle 4. Postsynaptic components were labeled with anti-DLG antibody (green) and presynaptic membranes were labeled with anti-HRP antibody (red). (A-C) The typical structure of individual and organized round boutons separated by links still present 4 h APF. At this stage, the anti-DLG staining surrounded the bouton membranes as a thick and fuzzy layer. (D-I) Between 5 and 7 h APF the overall NMJ morphology appeared disorganized. Some individual synaptic boutons (arrows in E) surrounded by an anti-DLG staining displaying filopodial structures can still be distinguished 5 h APF. Individual boutons could no longer be distinguished 7 h APF. As it is shown in H we could observe terminal axon ends detaching from the parent axon (arrows). (J-L) 9 h APF, the anti-DLG staining was mostly absent and only some anti-DLG-stained fragments were occasionally observed. At this stage, the presynapse was either absent or it appeared as a spherical structure as is shown here (asterisk in K). (M-O) At 10 h APF, NMJ similar than those seen as 9 h APF were observed. (P-R) Example of a remnant 12 h APF NMJ showing a small protrusion labeled by HRP at the presynapse and some DLG-labeled postsynaptic fragments. A-L, Bars, 20 µm. M-O, Bar, 25 µm.
(EPS)

Figure S2 **GFP membrane labeling of the motor neuron membranes is not disrupted.** (A-C) *OK6-GAL* driven *UAS-GFP* in motor neurons showed membrane continuity at late pupal stages. Motor neurons at muscle 4 are indicated by arrowheads. Bars, 30 µm. Genotype: *OK6-GAL4, UAS-GFP/+*.
(EPS)

Figure S3 **Microtubule disorganization and retraction but not degeneration takes place during NMJ remodeling (continued).** High magnifications and low exposures were used. Anti-Acetylated Tubulin (red) and anti-HRP (green) staining at 2 h, 5 h, 7 h, 10 h and 12 h APF. (A-J) Tubulin staining was present and continuous in motor neurons at all the stages analyzed. No Tubulin monomers were observed even at very late stages. In G and H, Tubulin-bundles corresponding to NMJ at muscle 4 were pointed by arrows. In I and J, the location of the NMJ was identified due to the presence of HRP-labelled presynaptic debris surrounding the nerve (arrowhead). Here again, we observe a continuous tubulin protrusion (arrow). Bars, 10 µm. Genotype: *MHC-mGFP-Shaker.*
(EPS)

Figure S4 **Disrupting macrophage function blocks NMJ dismantling (continued).** Percentage of 7 h APF NMJ showing arrested post and presynaptic dismantling at restrictive temperature in A: +/*shi* (n = 36), *Hml*/+ (n = 9) and *Hml/shi* (n = 39). B: +/*shi* (n = 36), *Pxn*/+ (n = 14) and *Pxn/shi* (n = 29). Expression of *shi^ts^* in phagocytes significantly increased the arrested post and presynaptic dismantling phenotypes. ***: P<0.001 (χ^2^ test). Genotypes: A: +/shi  =  +/*UAS-shi^ts1^*. Hml/+  =  *Hml-GAL4/+.* Hml/shi  =  *Hml-GAL4/UAS-shi^ts1^* B: +/shi  =  +/*UAS-shi^ts1^.* Pxn/+  =  *Pxn-GAL4*/+. Pxn/shi  =  *Pxn-GAL4*/*UAS-shi^ts1^.*
(EPS)

Figure S5 **ECR-B1 levels are decreased in** ***wit*** **mutant motor neuron.** (A-F) Ten composite confocal images of the ventral cord region. (A-C) Green represents anti-EVE staining and red represents anti-ECR-B1 staining in *yw* controls. EVE expressing motor neurons display also ECR-B1 expression (arrowheads). (D-F) Green represents *OK6-GAL4* driven GFP and red represents anti-ECR-B1 staining. GFP expressing motor neurons display also ECR-B1 expression (arrowheads). Bars, 50 µm. (G) Quantification of ECR-B1 signal in arbitrary units. Results are means and s.e.m. The anti-ECR-B1 signal presence in EVE-expressing larval motor neurons was quantified in controls (yw, n = 5) and *wit* mutant (*wit^−/−^*, n = 5) individuals. (H) Quantification of ECR-B1 signal in arbitrary units. Results are means and s.e.m. The anti-ECR-B1 signal present in GFP positive motor neurons was quantified in control (*OK6/GFP*, n = 5) and in *wit* dominant negative expressing (*wit^DN^*, *OK6/GFP*, n = 5) individuals. (D) Note that the ECR-B1 expression decreases in both *wit^−/−^*, and *wit^DN^* over-expressing motor neurons. The difference is highly significant in a *t*-test. ***: P<0.001 (χ^2^ test). Genotypes : (A-C) *y w^67c23^*, (D-F) *OK6-GAL4/2xGFP.* G : *yw*  =  *y w^67c23^*. *wit^−/−^*  =  *wit^A12^/wit^B11^*. H: *OK6/GFP*  =  *OK6-GAL4/2xUAS-GFP. wit^DN^, OK6/GFP  =  UAS-wit^DN^, OK6-GAL4/2xUAS-GFP.*
(EPS)
